# Occupational Sitting and Weight Status in a Diverse Sample of Employees in Midwest Metropolitan Cities, 2012–2013

**DOI:** 10.5888/pcd11.140286

**Published:** 2014-11-20

**Authors:** Lin Yang, J. Aaron Hipp, Christine M. Marx, Ross C. Brownson

**Affiliations:** Author Affiliations: Lin Yang, Division of Public Health Science, Department of Surgery, Washington University School of Medicine, St. Louis, Missouri; Christine M. Marx, Division of Public Health Science, Washington University School of Medicine, St. Louis, Missouri; Ross C. Brownson, Prevention Research Center in St. Louis, Brown School, Washington University in St. Louis, and Division of Public Health Science and Alvin J. Siteman Cancer Center, Department of Surgery, Washington University School of Medicine, St. Louis, Missouri.

## Abstract

**Introduction:**

Few studies have examined the association between occupational sitting and body mass index (BMI). There is a particular lack of evidence among diverse populations. The objective of this study was to quantify the association between self-reported occupational sitting time and BMI by sex and race, independent of levels of occupational and leisure-time physical activity.

**Methods:**

In 2012 and 2013, participants residing in 4 Missouri metropolitan areas were interviewed via telephone. The interview included questions on sociodemographic characteristics and time spent sitting at work. Multinomial logistic regressions were used to examine the association between occupational sitting and BMI between men and women and between black and white women.

**Results:**

Overall 1,891 participants (66.9% women, 29.5% black) provided complete data. Median daily time spent by both men and women in occupational sitting was 180 minutes (interquartile range, 30 to 360 minutes); most participants were overweight (32.3%) or obese (33.6%). After adjusting for potential confounders, we found that black women in 3 categories of sitting time (31–180 minutes, 181–360 minutes, and >360 minutes) were approximately 2.5 times as likely (*P* for trend, .02) to be obese as black women who reported sitting for 30 minutes or less, independent of occupational and leisure-time physical activity. This association was not seen among white women. No significant associations were found among men.

**Conclusion:**

Occupational sitting is associated with an increased likelihood of obesity among black women, independent of occupational and leisure-time physical activity. Areas of future research include evaluating associations among various occupations and industries, assessing the association in prospective cohorts, and exploring the feasibility of worksite interventions that target sitting.

## Introduction

Overweight and obesity are recognized as risk factors for various chronic diseases, and rates are increasing worldwide (1). The projected growth in chronic disease rates caused by trends in obesity during the next 20 years is expected to increase medical costs by $48 to $66 billion annually in the United States (2). Although men and women have similar overall obesity rates, the highest prevalence of obesity is among black men and women. Black women have higher obesity rates than black men (3). Physical inactivity may play an important role in the etiology of obesity (4). A growing body of research has focused on sedentary behavior in relation to health. Sedentary behavior refers to any waking activity characterized by an energy expenditure of 1.5 metabolic equivalents or less and a sitting or reclining posture (5). It is distinct from physical inactivity and can exist among physically active populations. For example, a desk-based office worker may accumulate 30 minutes of brisk walking before and after work but may spend up to 8 hours sitting at work.

Sedentary time is accumulated in various settings, such as in the home and workplace and during transit. Given that adults can spend 8 or more hours per day at work, workplaces may be an ideal setting to reduce sedentary time through implementation of worksite policies or changes to the physical work environment (6). Only a few studies have considered the association between occupational sitting and weight status. These studies are limited in that they included samples of women only (7,8) or lacked stratification between overweight and obese participants (7–9); no previous studies have examined potential differences by sex or race.

The objective of this study was to explore the association between self-reported occupational sitting and weight status by sex and race, also accounting for occupational and leisure-time physical activity, among a diverse sample of working adults in 4 Missouri metropolitan areas.

## Methods

### Study population and design

Participants were from the Supports at Home and Work for Maintaining Energy Balance (SHOW-ME) study (10), a cross-sectional study designed to understand how environmental and worksite policies influence the weight status of employees. Census tracts in 4 Missouri metropolitan areas (St. Louis, Kansas City, Springfield, and Columbia) were used for sampling. We excluded census tracts that had a population density in the lowest 10th percentile of the study areas and in which more than 50% of inhabitants were aged 15 to 24 years. Multistage stratified sampling procedures were used to sample individuals in 7 strata. We first stratified by metropolitan size (large [Kansas City and St. Louis] or small [Springfield and Columbia]). In each size stratum, we stratified by 3 levels of walkability using a walkability index (11) (low, <45th percentile; moderate, 45th–90th percentile; or high, >90th percentile) and by 2 levels of racial/ethnic diversity (low, >50% of the population was white; high, ≤50% of the population was white or ≥50% was Hispanic). Potential participants were reached and recruited using list-assisted telephone random-digit–dialing methods. Only one, and the first eligible, adult who volunteered to participate was sampled in each household. The response rate for interviews was 49%. During 2012 and 2013, 2,015 participants who met each of the following criteria were recruited: aged 21 to 65 years, employed outside of the home at 1 primary location, employed for 20 or more hours per week at 1 site with at least 5 employees, and not pregnant; and had no physical limitation that prevented walking or bicycling in the previous week. Recruited participants completed a telephone survey. The instrument was developed for this study and was based on existing self-reported and environmental assessment instruments with additional input from a questionnaire advisory panel composed of experts in survey development, nutrition and the food environment, physical activity, transportation, and worksite environmental intervention. Instrument development and telephone interview procedures have been detailed elsewhere (10). The study design was approved by the institutional review boards of Washington University in St. Louis and University of Missouri–Columbia. All participants provided informed consent.

### Measures

**Main outcome — body mass index (BMI).** Participants self-reported weight and height. BMI was calculated as weight in kilograms divided by height in meters squared and was categorized as underweight or normal weight (BMI <25.0), overweight (BMI 25.0 to <30.0), and obese (BMI ≥30.0) (12).

**Mean exposure — occupational sitting. **The telephone survey incorporated questions adapted from the Australian Longitudinal Study on Women’s Health (13) on the frequency and duration of sedentary behaviors that occur at work, home, and travel to and from work. Sitting time at work (occupational sitting) was determined by the following question: “Please estimate how many hours you spent sitting each day while at work.” Sitting time at work was recorded in hours and minutes and recoded as total minutes per day. Daily sitting time at work was categorized into the following quartiles: 30 minutes or less, 31 to 180 minutes, 181 to 360 minutes, and more than 360 minutes.

**Covariates and sociodemographic variables.** Participants self-reported age, sex, race, marital status, education, annual household income, employer size (number of employees), and a diagnosis by a physician of 3 chronic conditions (heart disease, diabetes, or cancer). Data on these characteristics were categorized.

**Worksite supports and policies.** Worksite supports and policies were determined by using 18 questions asking whether a set of policies or environments supporting physical activity were available at the worksite and whether the participants had ever used them. Possible responses were yes, no, or “do not know.” These questions show moderate to almost perfect reliability in measuring worksite policies and environments supporting physical activity (10). A summary score was derived by summing the number of responses in each category (yes, no, or do not know) and then dividing the summary score into 3 tertiles.

**Occupational and leisure-time physical activity. **Selected questions from the International Physical Activity Questionnaire (IPAQ) were administered to collect data on self-reported frequency and duration of occupational and leisure-time physical activities. IPAQ has been tested internationally for reliability (Spearman’s ρ ~ 0.8) and validated with objective measures (median ρ ~ 0.3); these values are comparable to values found in other validation studies of self-reported data (14). For each category (occupational physical activity and leisure-time physical activity), we dichotomized weekly minutes into less than 150 minutes per week (insufficiently active) or 150 or more minutes per week (sufficiently active), to determine whether participants were meeting recommended levels of physical activity (15) in either category of activity.

### Analyses

We used χ^2^ tests to assess differences between men and women by sociodemographic characteristics, employer size, worksite policy, occupational physical activity, leisure-time physical activity, and sitting time spent at work. Because of differences in weight status and physical activity behavior between men and women (16), we stratified our analyses by sex. Univariate logistic regression was conducted to quantify the associations between occupational sitting and categories of weight status and the association between occupational sitting and all covariates for men and women. Variables were entered into the final multinomial logistic regression models if they were significant at *P* < .10 in univariate analysis. For men, models were adjusted for age, marital status, annual household income, chronic condition, and occupational and leisure-time physical activity level; for women, models were adjusted for age, race, education, marital status, annual household income, chronic condition, and occupational and leisure-time physical activity level. Because of an insufficient number of men, we conducted the race-stratified analysis for women only. Because most women in the sample were white or black, we excluded other races. We then examined whether the association between occupational sitting and BMI differed for white women and black women. All analyses were performed using Stata version 12.0 (Stata Corp).

## Results

Of the 2,012 participants who completed the telephone survey, 1,891 (94.0%) participants reported their height and weight and the amount of time they spent sitting at work. The sample consisted of more women (66.9%) than men (Table 1). Most of the sample was white (63.0%) and overweight (32.3%) or obese (33.6%). Median daily time spent by both men and women in occupational sitting was 180 minutes (interquartile range, 30 to 360 minutes).

**Table 1:** Characteristics of Participants (N = 1,891) in the SHOW-ME Study by Sex, 4 Missouri Metropolitan Areas, 2012–2013^a^

Sociodemographic Characteristic	Men (n = 625), n (%)	Women (n = 1,266), n (%)	*P* Value
**Age, y**			
21–44	229 (36.8)	441 (34.9)	.55
45–54	207 (33.2)	412 (32.7)	
55–65	187 (30.0)	409 (32.4)	
**Race**			
White	446 (72.1)	746 (59.3)	<.001
Black	130 (21.0)	428 (34.0)	
Other	43 (6.9)	84 (6.7)	
**Education**			
High school degree or less	133 (21.3)	277 (21.9)	<.001
Some college	138 (22.1)	347 (27.4)	
College graduate	190 (30.4)	412 (32.5)	
Graduate degree	164 (26.2)	230 (18.2)	
**Marital status**			
Never married, divorced, separated or widowed	236 (37.8)	546 (43.2)	.02
Married or living with a partner	389 (64.2)	719 (56.8)	
**Body mass index, kg/m^2^**			
Underweight or normal weight (<25.0)	196 (31.4)	448 (35.4)	.004
Overweight (25.0 to <30.0)	234 (37.4)	377 (29.8)	
Obese (≥30.0)	195 (31.2)	441 (34.8)	
**Chronic condition^b^**			
No	516 (82.8)	1044 (82.7)	.93
Yes	107 (17.2)	219 (17.3)	
**Annual household income, $**			
<40,000	164 (27.8)	437 (36.4)	<.001
40,000 to <75,000	188 (31.9)	407 (33.9)	
≥75,000	237 (40.2)	357 (29.7)	
**Employer size, by no. of employees**			
5–49	227 (37.4)	364 (30.2)	<.001
50–199	151 (24.9)	426 (35.3)	
≥200	229 (37.7)	415 (34.4)	
**Occupational physical activity**			
Insufficiently active	295 (47.2)	681 (53.8)	.007
Sufficiently active	330 (52.8)	585 (46.2)	
**Leisure physical activity**			
Insufficiently active	260 (41.6)	660 (52.1)	<.001
Sufficiently active	365 (58.4)	606 (47.9)	
**Daily sedentary time at work, min**			
≤30	171 (27.4)	321 (25.4)	.61
31–180	169 (27.0)	327 (25.8)	
181–360	141 (22.6)	302 (23.8)	
>360	144 (23.0)	316 (25.0)	

Abbreviations: SHOW-ME, Supports at Home and Work for Maintaining Energy Balance.

a Percentages are based on the number of participants who responded to the question. Data were missing for the following categories: age, 2 men, 4 women; race, 6 men, 8 women; marital status, 1 woman; chronic condition, 2 men, 3 women; annual household income, 36 men, 65 women; employer size, 18 men, 61 women.

b Survey participants were asked whether they had been diagnosed by a physician as having any of 3 chronic conditions (heart disease, diabetes, or cancer); response options were yes or no.

Compared with women who spent 30 or minutes or less of daily sedentary time at work, women who spent 31 to 180 minutes were 1.53 times as likely to be obese, women who spent 181 to 360 minutes were 1.90 times as likely, and women who spent more than 360 minutes were 1.70 times as likely (*P* value for trend, .02) (Table 2). We found no association between occupational sitting and weight status among men.

**Table 2:** Adjusted Associations Between Occupational Sitting and Body Mass Index (BMI) Among Men (n = 625) and Women (1,266) in the SHOW-ME Study, 4 Missouri Metropolitan Areas, 2012–2013

Minutes of Sedentary Time Spent at Work	Overweight (BMI 25.0 to <30.0)			Obese (BMI ≥30.0)		
	n (%)	AOR (95% CI)	*P* Value	n (%)	AOR (95% CI)	*P* Value
**Men^a^**						
≤30	64 (27.4)	1 [Reference]	—	43 (22.1)	1 [Reference]	—
31–180	64 (27.4)	1.21 (0.71–2.08)	.48	56 (28.7)	1.65 (0.93–2.94)	.09
181–360	47 (20.1)	0.97 (0.53–1.78)	.91	54 (27.7)	1.88 (1.00–3.53)	.05
>360	59 (25.2)	1.43 (0.76–2.69)	.27	42 (21.5)	1.49 (0.75–2.96)	.26
Test for linear trend	.40			.18		
**Women^b^**						
≤30	89 (23.7)	1 [Reference]		108 (24.6)	1 [Reference]	
31–180	106 (28.2)	1.54 (1.02–2.32)	.04	109 (24.8)	1.53 (1.02–2.31)	.04
181–360	84 (22.3)	1.32 (0.85–2.06)	.22	115 (26.2)	1.90 (1.23–2.94)	.004
>360	97 (25.8)	1.50 (0.95–2.36)	.08	107 (24.4)	1.70 (1.08–2.67)	.02
Test for linear trend	.14			.02		

Abbreviations: SHOW-ME, Supports at Home and Work for Maintaining Energy Balance; AOR, adjusted odds ratio; CI, confidence interval; —, not applicable.

a Adjusted for age, marital status, annual household income, chronic condition, and occupational and leisure time physical activity level. Reference is underweight or normal weight.

b Adjusted for age, race, education, marital status, annual household income, chronic condition, and occupational and leisure time physical activity level. Reference is underweight or normal weight.

In the race-stratified analysis of women, we found an association between occupational sitting and weight status among black women but not white women (Table 3). Compared with black women who spent 30 minutes or less of sedentary time at work, black women who spent 31 to 180 minutes were 2.43 times as likely to be obese, black women who spent 181 to 360 minutes were 2.76 times as likely, and black women who spent more than 360 minutes were 2.53 times as likely (*P* value for trend, .02) 

**Table 3:** Adjusted^a^ Associations Between Occupational Sitting and Body Mass Index (BMI) Among White Women (n = 746) and Black Women (n = 428) in the SHOW-ME Study, 4 Missouri Metropolitan Areas, 2012–2013

Minutes of Sedentary Time Spent at Work	Overweight (BMI 25.0 to <30.0)			Obese (BMI ≥30.0)		
	n (%)	AOR (95% CI)	*P* Value	n (%)	AOR (95% CI)	*P* Value
**White women**						
≤30	33 (16.0)	1 [Reference]	—	49 (24.0)	1 [Reference]	—
31–180	62 (30.1)	2.11 (1.21–3.68)	.008	38 (18.6)	0.94 (0.53–1.66)	.84
181–360	50 (24.3)	1.76 (0.97–3.17)	.06	53 (26.0)	1.35 (0.76–2.39)	.31
>360	61 (29.6)	1.70 (0.93–3.10)	.09	64 (31.4)	1.15 (0.64–2.06)	.64
Test for linear trend	.22			.49		
**Black women**						
≤30	47 (33.8)	1 [Reference]	—	53 (25.6)	1 [Reference]	—
31–180	35 (25.2)	1.41 (0.67–2.97)	.37	61 (29.5)	2.43 (1.21–4.88)	.01
181–360	28 (20.1)	1.20 (0.51–2.78)	.51	55 (26.6)	2.76 (1.26–6.07)	.01
>360	29 (20.9)	1.75 (0.69–4.41)	.69	38 (18.4)	2.53 (1.04–6.12)	.04
Test for linear trend	.23			.02		

Abbreviation: SHOW-ME, Supports at Home and Work for Maintaining Energy Balance; OR, odds ratio; CI, confidence interval; —, not applicable.

a Adjusted for age, education, marital status, annual household income, chronic condition, and occupational and leisure time physical activity level. Reference is underweight or normal weight.

## Discussion

This study describes the association between self-reported occupational sitting and weight status among a working adult population in 4 Midwest metropolitan areas. We found a significant association between daily occupational sitting and an increased likelihood of obesity among women. Further stratification showed this association differed by race and was observed among black women but not white women. The association was consistent across different levels of sitting time.

The proportion of obese participants (33.6%) in our sample is similar to the proportion found in a recent national study (35.7%) (3). Few studies have reported on the association between occupational sitting and weight status among a working population of men and women. Our findings are consistent with those of a prospective study of women (7), which reported a significant trend of a 5% increase in obesity with each 2-hour-per-day increment in occupational sitting, independent of a total physical activity metabolic equivalent score. In that population, the only significant difference found among 4 categories of sitting time was between women who sat more than 40 hours per week and women who sat less than 1 hour per week (7).

Our findings are not consistent with those of an Australian study, which reported that men who sat more than 6 hours per day were 1.92 (95% confidence interval, 1.17–3.71) times as likely to be overweight or obese as men who sat for less than 45 minutes per day, and no association was found among women (9). The difference in reported associations may have been due to differences in characteristics between the 2 samples. The proportion of participants of normal weight in the Australian sample (45.5%) was higher than that in our sample (34.1%). In addition, the Australian study (9) grouped overweight and obesity together; our analysis separated overweight and obesity.

Another study, conducted among older Australian women, found higher BMI among women who self-reported insufficient leisure-time physical activity and had occupations that involved sitting for most of the work day than among women in 3 other groups — those reporting insufficient self-reported leisure-time physical activity and no occupational sitting, those reporting sufficient self-reported leisure-time physical activity and mostly occupational sitting, and those reporting sufficient self-reported leisure-time physical activity and no occupational sitting (8). Although the analysis used a different approach for treating the BMI variable than ours did, the findings on the association between occupational sitting and weight status are consistent with ours. Our analysis further demonstrated this association is independent of the level of leisure-time physical activity of women.

The lack of association between occupational sitting and weight status among men might be explained by the differences between men and women in physical activity preferences (16). Men are more active in leisure-time physical activity than women (16), and women tend to do less vigorous and more moderate activity compared with men (17). US guidelines suggest that adults accumulate at least 150 minutes of moderate (eg, brisk walking) or 75 minutes of vigorous intensity physical activity (eg, jogging or running) or an equivalent combination of both per week (15). We used categorical variables for occupational and leisure-time physical activity to indicate whether participants were meeting CDC recommendations by combining time spent in both moderate and vigorous physical activity in each domain. Therefore, men who meet CDC recommendations may be expending more energy than women who meet CDC recommendations by participating in mostly vigorous rather than moderate activity (18). The stronger associations between sitting time and obesity among black women may be related to confounders or interactions with factors such as the home food environment, perceptions on body ideals (19), or lower resting metabolic rates (20).

To the best of our knowledge, this is the first study to examine differences in the association between occupational sitting and weight status among black women and white women. The main strengths of the study are the large sample of employed men and women and the inclusion of comprehensive sociodemographic and worksite characteristic variables. Unlike studies that used only nonobese and obese as categories of weight status (7,9), we used 3 categories: normal weight, overweight, and obese. There is a strong case for separating overweight from the nonobese category, given the health consequence of being overweight but not obese (1,21,22). Additionally, using 3 categories for weight status will facilitate comparisons among future studies.

This study has several limitations. The number of missing values for annual household income and employer size is high compared with the number of missing values for other covariates. However, when we conducted a sensitivity analysis, excluding all missing values, we found odd ratios of 1.48 (31–180 minutes of sedentary time), 1.93 (181–360 minutes of sedentary time), and 1.63 (>360 minutes of sedentary time) among women of both races (*P* value for trend, .03). Among black women, the sensitivity analysis resulted in odd ratios of 2.52 (31–180 minutes of sedentary time), 2.87 (181–360 minutes of sedentary time), and 2.72 (>360 minutes of sedentary time) (*P* value for trend, .01). We were not able to conduct race-stratified analyses for men. The largest effect sizes in the sex-specific model were used to perform the post-hoc sample size calculation with at least 80% power. We found that men were underpowered because of an insufficient number of black men (n = 144). Because of its cross-sectional design, our study cannot determine a causal relationship between occupational sitting and weight status. The measurement instruments used in this study are reliable (10), although our data, including data on height and weight, were self-reported and may be subject to response bias (23). Finally, because of the sampling strategy and the restricted geographic area, the generalizability of the findings may be limited.

Changes in population-level patterns of physical activity have contributed to the rise in obesity (24). Sedentary behavior is also a possible independent contributor. However, the causal pathway between sedentary behavior and obesity is unclear: are overweight people more likely to accumulate sedentary time, or are greater amounts of sedentary time leading to declines in energy expenditure and increases in weight? Despite evidence suggesting various levels of association between occupational sitting and weight status in the United States and Australia, the direction of the association is consistent in showing that a higher likelihood of obesity is related to prolonged occupational sitting (7–9). However, only one study (7) used data from a prospective cohort. Future research should include longitudinal data from racially and ethnically diverse populations to explore possible causal pathways and examine factors associated with differences by race and ethnicity. 

Methods for measuring sedentary behavior are not well developed. Self-reported measures of sedentary behavior focus on television viewing, whereas objective measures, such as accelerometers, have limited capacity to capture data on body posture or physical activity settings (leisure-time, occupational, transportation, etc.) (25). The development of a reliable and valid measurement for context-specific sedentary behavior (occupational sitting in this case) is a challenge but would enable future studies to provide stronger evidence to inform intervention design among public health researchers and practitioners, worksite supports and policy planners, and other stakeholders.

More attention should be paid to developing interventions that reduce levels of sedentary behavior in a worksite setting (26). Most physical activity intervention studies focus on promoting physical activity. The UK physical activity guidelines recommend the same level and amount of physical activity recommended by CDC, but they also recommended in 2011 for the first time that adults minimize sitting time (27). Breaks in sedentary time improve metabolic risk biomarkers (28). Reducing the amount of sitting time and interrupting sitting time by active breaks is recommended, even for adults who meet recommended levels of physical activity (27,29). In addition, adults with sedentary occupations should aim to maintain a healthy diet to avoid weight gain. To develop such interventions in a worksite setting, better understanding of the correlates and determinants of occupational sitting behavior is required. Feasibility studies, ideally using mixed methods and incorporating robust objective measurements and qualitative interviews, may be useful in exploring new worksite approaches, such as using standing desks.

In this sample of men and women working in Midwest metropolitan areas, women who sat for more than 30 minutes each working day were more likely to be obese than women who sat 30 minutes or less, independent of occupational activity and leisure-time physical activity. This association was stronger for black women than for white women. There is also probably a trend for an increased risk of overweight with increased occupational sitting. We found no such association among men. Further studies are needed to investigate a possible causal relationship between occupational sitting and weight status. Meanwhile, intervention feasibility studies are needed to examine the effectiveness of worksite policy and environmental modification in reducing the amount of sedentary behavior at work.
